# Recent Advances in Prodigiosin as a Bioactive Compound in Nanocomposite Applications

**DOI:** 10.3390/molecules27154982

**Published:** 2022-08-05

**Authors:** Rafael G. Araújo, Natalia Rodríguez Zavala, Carlos Castillo-Zacarías, Mario E. Barocio, Enrique Hidalgo-Vázquez, Lizeth Parra-Arroyo, Jesús Alfredo Rodríguez-Hernández, María Adriana Martínez-Prado, Juan Eduardo Sosa-Hernández, Manuel Martínez-Ruiz, Wei Ning Chen, Damià Barceló, Hafiz M.N. Iqbal, Roberto Parra-Saldívar

**Affiliations:** 1 Tecnologico de Monterrey, Institute of Advanced Materials for Sustainable Manufacturing Monterrey, Monterrey 64849, Mexico; 2 Chemical & Biochemical Engineering Department, Tecnológico Nacional de México-Instituto Tecnológico de Durango (TecNM-ITD), Blvd. Felipe Pescador 1830 Ote. Durango, Durango 34080, Mexico; 3 Universidad Autónoma de Nuevo León, Facultad de Ingeniería Civil, Departamento de Ingeniería Ambiental, Ciudad Universitaria S/N, San Nicolás de los Garza 66455, Mexico; 4 Tecnologico de Monterrey, School of Engineering and Sciences, Monterrey 64849, Mexico; 5 School of Chemical and Biomedical Engineering, Nanyang Technological University, 62 Nanyang Drive, Singapore 637457, Singapore; 6 Department of Environmental Chemistry, Institute of Environmental Assessment and Water Research, IDAEA-CSIC, 08034 Barcelona, Spain; 7 Catalan Institute for Water Research (ICRA-CERCA), Parc Científic i Tecnològic de la Universitat de Girona, Edifici H2O, 17003 Girona, Spain; 8 Sustainability Cluster, School of Engineering, UPES, Dehradun 248007, India

**Keywords:** bionanocomposites, prodigiosin, pyrrolic compounds, *Serratia marcescens*, biorenewable resources

## Abstract

Bionanocomposites based on natural bioactive entities have gained importance due to their abundance; renewable and environmentally benign nature; and outstanding properties with applied perspective. Additionally, their formulation with biological molecules with antimicrobial, antioxidant, and anticancer activities has been produced nowadays. The present review details the state of the art and the importance of this pyrrolic compound produced by microorganisms, with interest towards *Serratia marcescens*, including production strategies at a laboratory level and scale-up to bioreactors. Promising results of its biological activity have been reported to date, and the advances and applications in bionanocomposites are the most recent strategy to potentiate and to obtain new carriers for the transport and controlled release of prodigiosin. Prodigiosin, a bioactive secondary metabolite, produced by *Serratia marcescens*, is an effective proapoptotic agent against bacterial and fungal strains as well as cancer cell lines. Furthermore, this molecule presents antioxidant activity, which makes it ideal for treating wounds and promoting the general improvement of the immune system. Likewise, some of the characteristics of prodigiosin, such as hydrophobicity, limit its use for medical and biotechnological applications; however, this can be overcome by using it as a component of a bionanocomposite. This review focuses on the chemistry and the structure of the bionanocomposites currently developed using biorenewable resources. Moreover, the work illuminates recent developments in pyrrole-based bionanocomposites, with special insight to its application in the medical area.

## 1. Introduction

**Figure 1:**
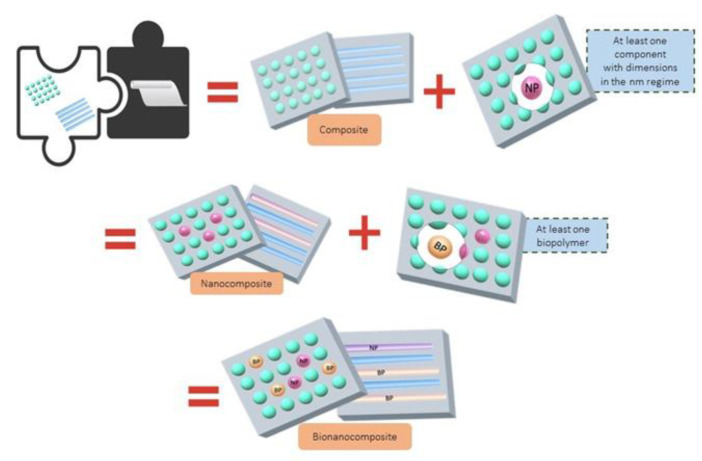
Bionanocomposites: Polymeric materials produced by bioprocesses (plants or microorganisms) based on nanostructure of materials derived from self-organization.

Bionanocomposites is a novel class of nanometric materials that has gained a lot of interest lately as it is a technology that integrates many fundamental characteristics to create new materials with unique properties. Bionanocomposites, also known as “nanobiocomposite”, “green composite”, and/or “bio-based plastics”, are similar to nanocomposites, but they have critical differences, such as their applications, functionalities, methods of preparation, compounds, properties, biodegradability, and biocompatibility, among others [1,2,3,4,5]. They are made up of a natural polymer (biopolymer, BP) and an inorganic compound at a nano scale (nanoparticle, NP), as illustrated in Figure 1.

**Figure 2:**
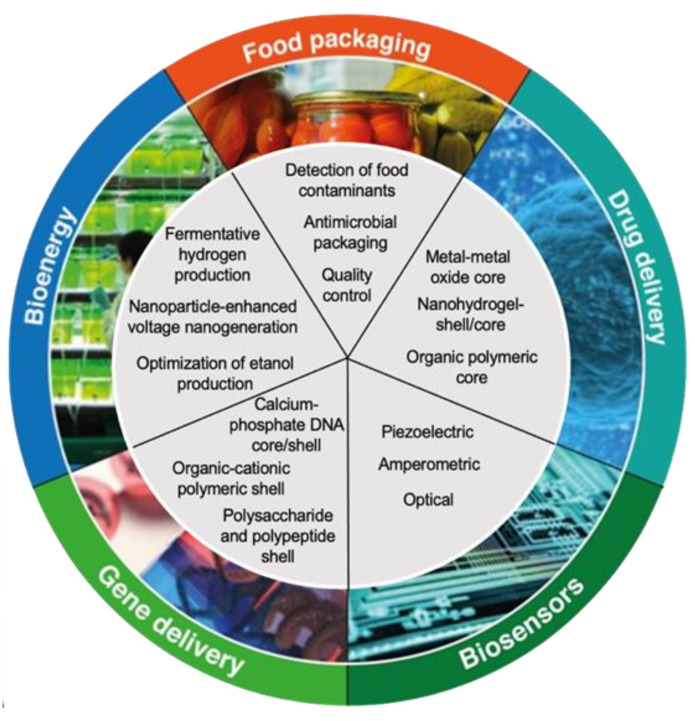
Bionanocomposites: Versatility and their applications in different industries.

The shape of the nanoparticle is essential in the formation of the nanocomposite, it can be divided into different categories, such as nanorods, nanofibers, nanotubes, nanoparticles, and nanoplates. Polymer nanocomposites (PNCs) have been of interest due to their high availability, cost-effectiveness, and ease of production. PNCs are used more in the industry due to their lightness, ease of production, and their malleable nature. Unlike metals or ceramics nanocomposites, polymer-based nanocomposites have low strength; however, polymers can be filled with inorganic, synthetic, or natural compounds, increasing their resistance, mechanical strength, and some other characteristics [1,2,3,4,5,6]. Polymer-based nanocomposites have many benefits, according to several authors [7]. For instance, polymer-based nanocomposites are less porous than common plastic, so they are perfect to use in the protection of medical instruments, films, as a drug delivery system and so on. Additionally, bionanocomposites have great potential for various applications ranging from food packing, drug delivery, biosensors, gene delivery, and bioenergy production [8,9,10,11,12,13,14], as shown in Figure 2.

For many biomedical applications, different bioactivities can be achieved through co-polymerization with natural polysaccharides, such as starch, cellulose, and chitosan, as well as with some proteins, such as albumin, legume and gelatin, which are widely used for the preparation of nanostructures for delivery of different drugs derived from its non-toxicity, stability, low size, and biodegradability. Another approach of interest is the incorporation of natural bioactive compounds in nanoencapsulated systems or in nanoemulsions that can be released in a controlled manner and generate a specific bioactivity [11].

Prodigiosin is a natural red pigment that is obtained through a select group of Gram-negative bacteria of the *Pseudomonas*, *Streptomyces*, and *Serratia genera*. Only a few Gram-positive bacteria, such as *Streptoverticillium rubrireticuli* and *Streptomyces longisporus*, have been described with the ability to produce prodigiosin. It has been reported that a greater number of Gram-negative prodigiosin producing bacteria, compared to Gram-positive organisms, can be found in the literature, such as *Pseudomonas magnesloruba*, *Vibrio psychroeryrhrous, Serratia rubidaea, Vibrio gazogenes, Alteromonas rubra, Rugamonas rubra, Serratia sp, and Serratia marcescens*; the latter being the characteristic microorganism in pigment production, it has also been identified that both groups of bacteria produce prodigiosin as a secondary metabolite [14,15,16,17,18,19,20,21].

Prodigiosin ($C_{20}H_{25}N_{3}O)$ and some of its analogs, such as undecylprodigiosin ($C_{25}H_{35}N_{3}O)$, cycloprodigiosin ($C_{20}H_{23}N_{3}O)$, and metacycloprodigiosin ($C_{25}H_{33}N_{3}O)$, present important activities of great interest to the pharmacological industry, due to their antimicrobial, antifungal, antiprotozoal, antimalarial, anticancer, immunosuppressant, and antiviral activities. Prodigiosin is considered as the most important candidate for cancer treatment due to its low toxic effect over normal cells, and it has application as a natural colorant. Other possible applications that have recently emerged is the use of the molecule as a pH indicator, a UV ray protector, an autofluorescence product, and its application as a controlling agent in the formation of biofilm [16,22,23,24,25,26]. So far, the factors that have been identified that affect the production of prodigiosin are the type of microorganism, culture medium, incubation time, temperature, nitrogen, and phosphorus concentration, inorganic salts, and pH [15,20,27,28,29]. Different fermentation strategies have been applied to produce prodigiosin, as well as strategies for statistical optimization, genetic manipulation and cloning, optimized cultures with gamma rays, co-cultures, and even cultures mediated by elicitation.

In this review, we focus on the different production strategies and bioactivities of prodigiosin as a compound of interest for the development of sustainable and renewable bionanocomposites with special attention to its application in the medical area.

## 2. Pyrrolic Compounds Produced by Microorganisms: *Serratia marcescens*

Secondary metabolites from microorganisms are one of the principal sources of active molecules that can be used in food, health, and biotechnology areas. Pyrrolic compounds are ring-structured and electron rich that can form hydrogen bonds and coordinating metals [30]. Pyrrolic compounds have several applications, and they have been used in antibiotic, anti-inflammatory, antitumor, and anti-cholesterol drugs. Moreover, these pyrrolic molecules have applications in polymerization and metallurgical reactions and as corrosion inhibitors and preservative agents [31]. Pyrrolic compounds are produced naturally by different bacterial strains found in nature, *Serratia marcescens* being the principal producer of these compounds. *Serratia marcescens* is a Gram-negative rod-shaped facultative bacterium, from the Enterobacteriaceae family. According to Hardjito et al., there are two types of *Serratia marcescens*, the unpigmented and the pigmented strains, the latter is characterized by its ability to produce the red pigment called prodigiosin [32]. Since it is a facultative bacterium, the pigment can be produced under aerobic and anaerobic conditions [33]. There are 10 species of the genus *Serratia*, however, only 3 of these can produce prodigiosin: *S. plymuthica*, *S. rubidaea*, and *S. marcescens*. The production of prodigiosin by the bacteria *Serratia marcescens* depends on different conditions, such as availability of inorganic phosphate, the composition of the medium, as well as the pH, temperature, and natural components of the growth media [34].

### Structure of Prodigiosin

**Figure 3:**
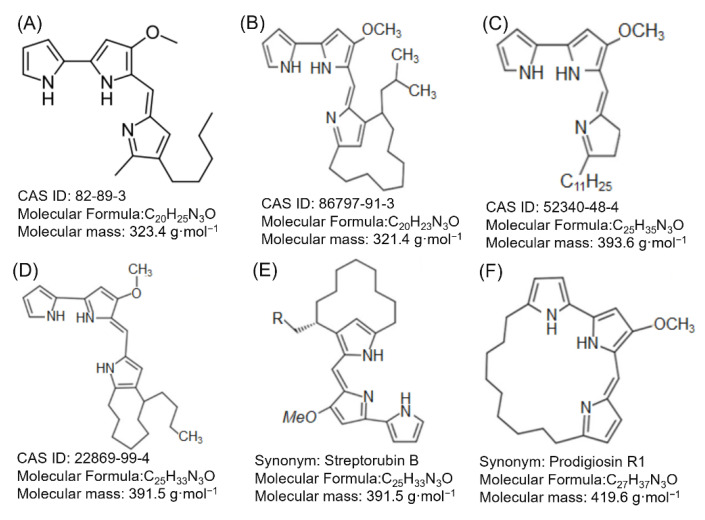
Examples of natural prodiginines: Prodigiosin (**A**), Cycloprodigiosin (**B**), Undecylprodigiosin (**C**), Metacycloprodigiosin (**D**), Butyl-meta-cycloprodigiosin (**E**), and Prodigiosin R1 (**F**).

Prodigiosin is part of the family of prodiginine, secondary alkaloids with a chemical structure of tripyrrol, with two rings joined and the third linked through a methane bridge that the molecules that are part of this family are differentiated between. Due to the size and arrangement of the branch in the last-named ring, this family is made up of Prodigiosin, Undecylprodigiosin, Cycloprodigiosin, Methacycloprodigiosin, Butyl-meta-cycloprodigiosin, and Prodigiosin R1 (Figure 3). These structures are characterized by being a part of a pigment that has aroused interest due to its antitumor, immunosuppressive, and anticancer activity. The molecular formula for prodigiosin is $C_{20}H_{25}N_{3}O$, and its molecular weight is 323.4 g/mol (232.44 Da); it can be dissolved in non-polar compounds, such as chloroform, methanol, acetonitrile, and dimethylsulfoxide, and it can partially be dissolved in alcohol and ether [20,35,36].

In total for *Serratia* sp. ATCC 39006 has identified a group of genes responsible for the biosynthesis of prodigiosin ranging from PigA to PigO, with PigC being the most important. The genes involved in MBC biosynthesis have also been found, which are PigI, PigG, PigA, PigJ, PigH, PigM, PigF, and PigN; and, for the biosynthesis of MAP, PigD, PigE, and PigB [37,38,39]. All this enzymatic complex that is activated in the production of prodigiosin is interconnected and stimulated by the carbon source, availability of phosphate, stationary phase, and cyclic signaling-di-GMP. It is believed that the synthesis of prodigiosin is accompanied by the synthesis of biosurfactants to improve its defense mechanism against other microorganisms or when it reaches high cell density through quorum sensing [40].

Investigations through a quorum sensing modulator effect in *Serratia marcescens* MTCC 97 shows that the stimulation by ultrasound at a frequency of 500 and 600 Hz stimulates the production of prodigiosin without affecting cell growth; meanwhile, frequencies of 400 and 700 Hz increase the production of prodigiosin but directly affect cell growth, which could allow future study of the modulating effect of the enzyme complex and gene expression on the production of prodigiosin [41]. The importance of one of the signaling molecules in the mechanisms of quorum sensing and therefore in the production of prodigiosin, using the hydrophobic cavity and the hydrophilic surface layer of the α-cyclodextrin immobilized in calcium alginate, has been found to manage to capture the extracellular signaling molecule N-acyl homoserin lactone (AHL), thereby inhibiting prodigiosin production by *S. marcescens* AS-1. It has also been found that for *Streptomyces coelicolor* prodigiosin plays a fundamental role in the regulation of programmed cell death, since prodigiosin has shown hyper-accumulation in the dead mycelia of the strain and a study carried out by suppressing the prodigiosin genes showed greater viability of the filaments, with respect to the non-mutated strain [42].

In addition, it has been found that its synthesis is directly related to the high production of ATP during the stage of cellular delay, just as pigmented cells reproduce at a higher rate and accumulate at a higher ATP velocity than non-pigmented cells, showing that ATP levels are direct regulators of prodigiosin production [43]. The use of glucose as a carbon source also inhibits the production of pigment, but this type of inhibition is still unknown since it would be thought that high levels of glucose would induce a high production of ATP and the high concentration of ATP in turn would induce pigment production. It was also thought that cAMP was a positive inducer of prodigiosin production, supported by the hypothesis that at low glucose levels cAMP levels would be elevated, but it has been found that both high and low glucose levels of cAMP inhibit the production of prodigiosin, so there is still a lack of knowledge about the mechanism of repression of glucose on pigment production [44]. On the other hand, the addition of amino acids with structures such as pyrrole—as in the case of proline, histidine, ornithine, aspartic acid, and glutamic acid—induce the production of prodigiosin, which suggests a direct incorporation in the metabolic route for synthesis of MBC and MAP [32].

## 3. Prodigiosin Producing Bacteria

The production of the red pigment as mentioned above is limited to an exclusive group of Gram-negative bacteria and in a smaller proportion to Gram-positive bacteria, with *Serratia* as the most important genus in the production of prodiginins, and *Serratia marcescens* as the species with greater number of reports in the production of prodiginins, specifically prodigiosin [45]. Other microorganisms belonging to the genus *Streptomyces* have been reported for their ability to produce prodiginins (Figure 3), such as Butylcycloheptylprodigiosin, Metacycloprodigiosin, Prodigiosin R1, and Undecylprodigiosin, with a higher production report of the *Streptomyces coelicolor* species [26,46]. *Pseudoalteromona* sp 1020R species have also been reported with the ability to produce up to four different prodiginins (2-Methyl-3-Butylprodiginine, 2-Methyl-3-Pentylprodiginine, 2-Methyl-3-Hexylprodiginine, and 2-Methyl-3 -Hytylprodiginine) [47]. Some microorganisms have been reported in the last decade with the ability to produce prodiginins (Table 1). It should be emphasized that many of the reports found in the literature to date from a red pigment identified as prodigiosin—which were based on the spectrum of UV-VIS absorption in basic and alkaline conditions, this spectrum can range between 470 and 537 nm in these conditions, frequently reaching a maximum absorption peak at 535 nm—this information is not sufficient for the identification of prodigiosin, since its analogs present the same spectrum of absorption. To be able to exactly identify the type of prodiginine requires techniques such as mass spectrometry or nuclear magnetic resonance. Another of the confusions that arises around prodigiosins is that they are a family of compounds of red color and that compounds such as Uncedylprodigiosina, Cycloprodogidiosin, Cyclononylprodigiosin, and Butylcycloheptylprodigiosina among others, are a part of this family, when the family to which prodigiosin and its previously named analogues really belong is the family of prodiginin, which has the characteristic skeleton pyrrolyl dipyrromethene [25,31].

### 3.1. Prodigiosin Production

As the biotechnological industry expands, techniques for microbial cultivation and growth become more sophisticated and optimal. However, at the present, large-scale production of prodigiosin remains as an opportunity area for this technology since current strategies and solutions for its obtention are expensive and complex [60]. As previously mentioned, pigmented strains from the *Serratia* genus are often exploited as a scalable source to produce prodigiosin and, therefore, its utilization for these purposes has been highly reported, representing a promising solution for the current state-of-the-art of this concern [61].

Several physical, chemical, and biological methods have been tested in order to improve the synthesis of the pigment in the gram-negative bacteria. A study carried by Gondil et al., aimed to determine the response of the wild strain *S. nematodiphila* RL2, which was previously isolated and identified, to a wide number of physicochemical parameters, such as culture medium used, carbon source, nitrogen source, pH, temperature, fermentation time, and metal ions present during fermentation, for the optimization of biomass generation and the enhancement of prodigiosin production. For initial experiments, nutrient broth, LB broth, tomato juice broth and yeast potato dextrose broth culture mediums were tested in a 50-h fermentation, at 35 °C at an initial pH 7, observing a major production of the red pigment in nutrient broth, which was detected at up to 0.49 mg/ mL, 17.9% higher than the next best result, obtained with yeast potato dextrose, which achieved a production of prodigiosin of 0.39 mg/mL. Nutrient medium was, hence, selected to perform the further experiments during this study. To select the more suitable carbon source during fermentation, 9 carbon species were explored: sucrose, galactose, glucose, maleic acid, ammonium acetate, citric acid, glycerol, sodium oxalate and lactose, at a concentration of 1% *w*/*v* in all treatments. As a result, prodigiosin was generated in higher concentrations using lactose as a carbon source, achieving 0.52 mg/mL of pigment produced. In a similar experiment, six different nitrogen sources were compared, determining that an optimal production of the pigment (0.6 mg/mL) can be achieved with yeast extract at 1% *w*/*v*. Likewise, 8 different initial pH values in the range from 3 to 10 were studied, observing the best results at pH 6 and 7 (0.6 mg/mL). A similar result was reported during the experiments to estimate the optimal temperature for prodigiosin production, in which, among 5 temperatures, the higher pigment concentration was detected at up to 0.59 mg/mL, at 35 °C. In addition, during the tests with metal particles, authors demonstrated that the presence of cobalt chloride and mercuric chloride (100 mM in each case) may severely down-regulate the synthesis of prodigiosin in the gram-negative strain studied, reducing its formation up to 0.16 and 0.17 mg/mL, respectively, even though fermentation is carried under the optimal parameter previously established. On the other hand, the presence of uranyl acetate during cultivation rocketed the pigment production up to 0.76 mg/mL [54].

In another study performed by Bhagwat and Padalia et al., authors successfully intended to reduce the costs of bioprocesses involved in the biotechnological production of prodigiosin by utilizing a variety of residues from the food industry as substrates for the commercial strain S. *marcescens* ATCC 13880, in a circular economy model [62]. In their research, authors tested the potential of different oil-based residues to function as a nutrient source for fatty acids obtention for the mentioned strain in order to design a model for waste revalorization while optimizing the cost for microbial prodigiosin production. Powders from three different oil sources, such as peanut, sesame and mustard were prepared for its addition to nutrient agar to run a fermentation performed under a series of physical and chemical parameters previously optimized in this study. In all cases the prodigiosin production increased in comparison with a treatment with non-treated cultivations, however, the best performance was observed in treatments with oil from peanut seed powder 4% *w*/*v*. Considering the obtained results, authors design an optimized culture medium based on nutrient agar with the next additives: casein hydrolysate at 1% *w*/*v* as nitrogen source, sucrose at 2% *w*/*v* as carbon source, and peanut seed powder at 4% *w*/*v*. Likewise, the final optimal fermentation was performed with an initial pH 7.5, at 28 °C during 72 h, producing 3.5 mg/mL of prodigiosin. In a similar series of experiments, Xia et al., utilized residues from a kitchen waste handle plant as an innovative nutrient substrate for the optimization of prodigiosin production using a wild strain of *S. marcescens* as biological model. Ideal operation parameters for fermentation, such as initial pH, temperature, carbon source, nitrogen source and agitation speed, were identified as well. Kitchen wastes were tested as substrates at 7 different concentrations between 20 and 50 g/L, where authors concluded that 35 g/L was the most appropriate substrate concentration, enhancing prodigiosin generation at >200 mg/mL [63]. 

Regarding chemical methods for the optimization of prodigiosin production, Chilczuk et al., designed a culture system in which the commercial strain *Serratia* sp. ATCC 39006 is in interaction with four ambigols (ambigol A, ambigol B, ambigol C and ambigol D), extracted from the cyanobacterium *Fischerella ambigua*. It was found out that ambigol C at 15.6 μM may enhance prodigiosin synthesis by around 4 times in comparison with control treatments [17]. According to transcriptomic analysis, this molecule improves the expression of genes involved in proline consumption, which has been proved to be a potential inductor of the synthesis of the natural red dye [63]. On the other hand, ambigols A and D demonstrated an antimicrobial activity in the presence of the gram-negative strain. 

Molecular techniques have been also reported, for instance, Sun et al. successfully developed an efficient protocol for the rocketing of the synthesis of prodigiosin by performing genetic and metabolic engineering on a wild strain of *S. marcescens* [64]. Their research focused on one of the thermoregulator systems (TSs) of the strain, involved in the silencing of the expression of *pig* proteins that are part of the mechanism of synthesis of prodigiosin, particularly, on the *cpx* complex, a two-component regulatory system, which operates under the activity of a sensor histidine kinase (CpxA), and a transcriptional regulatory CpxR protein [63]. This TS activates under a variety of external stress conditions, such as the presence of metal ions with antimicrobial activity, pH changes and high temperatures (>37 °C), resulting in a down-regulation of the expression of prodigiosin and other by-products [63,64,65,66]. The molecular mechanism of action of this system relies in the detection of such stress factors by the CpxA protein, followed by the phosphorylation of aspartic acid residues located along the structure of the CpxR, allowing its attachment to gene promoters that encodes *pig* proteins, and impeding their transcription [64,67]. To avoid the effects of this TS, authors developed two transformed strains of *S. marcescens* JNB 5-1 with suppressed *cpxA* and *cpxR* genes, respectively, and evaluated their capability to enhance the prodigiosin synthesis in comparison with a wild strain. Authors found out that the modified strain with suppressed *cpxR* genes were able to synthesize up to 76% more prodigiosin than the wild strain. Transcriptomic assays also showed a significantly higher expression of *pig* genes in this strain. The fermentation was carried at 30 °C, in LB culture medium in all treatments. Further experiments in this study aimed to evaluate the influence of temperature in the expression of *cpxA* and *cpxR* genes in the wild strain, comparing the transcription level of these genes at 30 °C and 37 °C in LB medium, and concluding that, in both cases, this was up to 6 times higher at 37 °C after almost 40 h of fermentation.

### 3.2. Prodigiosin Separation

Even though the most relevant factor behind the high values of prodigiosin in markets is due to the difficulty to obtain higher production yields during large-scale fermentations [63], the development of novel downstream methods is still an important subject in the generation of literature regarding the bioprocess of the pigment. According to Sun et al., most of the current state-of-the-art of methodologies for extraction and purification of prodigiosin are based on solvents, and large amounts of solvent are often required to improve the extraction yield of the process [68]. However, using organic solvents for large-scale extractions may be expensive and, in some cases, highly polluting [69]. At the present, alternative technologies have emerged to avoid such problems involved with traditional prodigiosin separation. In their research, Khanam and Chandra, determined that it is possible to obtain higher extraction yields for prodigiosin from *S. marcescens* by using inorganic solvents such as HCl, however it is possible to obtain similar yields with organic, less contaminating solvents such as ethanol when performing extractions at high temperatures (60 °C). Authors also evidenced the contribution of using physical cell-disruption techniques during extraction, such as ultrasonication, with which extraction yields may be doubled in comparison with using solvent-based (ethanol) extractions. Additionally, a degree of extraction of prodigiosin almost 100% was achieved by utilizing ultrasonication, while reducing the non-extracted fraction to almost 0% [70].

As another example of innovative separation techniques for prodigiosin produced by microorganisms, Arivizhivendhan et al., proposed a successful methodology for a solvent-free prodigiosin extraction by using iron oxide particles, adsorbing the pigment on their surface, in an integrated continuous fermentation system [58]. After their synthesis, Fe_3_O_4_ particles were functionalized using diethanolamine to work as a cross-linker between the metal particle and the prodigiosin. Subsequently, functionalized metal particles and pigment molecules interacted during 30 min at 150 rpm, and the resultant complex is then recovered from both stirred batch reactors used in this study. At this point, prodigiosin can be isolated from metal particles by using acetone, while this solvent can be easily removed by evaporation. Authors reported a recovery yield of 98% by using the functionalized Fe_3_O_4_ particles, meaning a recovery of prodigiosin of around 1046 mg/L. In addition, the synthesized iron oxide complex was proved to be highly reusable since similar adsorption yields for prodigiosin were observed during their second extraction cycle.

## 4. Biological Activity of Prodigiosin

Prodigiosin is characterized by having applications of great interest to the pharmacological industry. It has been shown in laboratory tests that prodigiosin has a good biological activity, such as antibacterial, antifungal, and antiviral. The benefit of having a bacterium with a secondary metabolite that has biological activity relies on the cost-effective production and the large amount of uses that this can have, not only in the pharmacological area but also in food preservatives, such as food coloring agents, among others.

### 4.1. Antimicrobial

This tripyrrole red pigment, has a notable antibacterial activity against Gram-negative bacteria such as Escherichia coli, Aeromonas hydrophila, Klebsiella pneumoniae, Proteus vulgaris, Proteus mirabilis, Pseudomonas aeruginosa, Salmonella enteritidis, and Salmonella Typhimurium [24,71,72], in addition to Gram-positive bacteria such as Staphylococcus aureus as well as its methicillin resistant strain, MRSA, Bacillus cereus, Corynebacterium glutamicum, Enterococcus faecalis, Enterococcus faecium, and Listeria monocytogenes [71,73,74,75]. Table 2 shows antibacterial activity reported in different studies. The forms of prodigiosin used in the studies varied, purified pigment was the most used, while a few used stained textiles and others crude rhizosphere extract. Among the different parameters for antimicrobial activity: the ATCC 100 value measures antibacterial activity in textiles; the antibacterial rate is the percentage difference between the number of colonies of the organism in dyed and undyed experiments; the IC_50_ is the concentration needed to reduce cellular viability to 50%; the maximum zone of inhibition measures the halo left by the compound in a petri dish seeded with a certain microorganism and the minimum inhibitory concentration that reports the smallest amount of the compound needed in order to affect cellular viability. Ren et al. observed the effect of pH on the antibacterial effect of prodigiosin dyed textiles with the highest toxicity at pH 8.1, this is possibly due to the fact that at this pH the solubility of the dye increases leading to more pigment being loaded on the textile [75]. The general mechanisms of prodigiosin microbial action are the cleavage of genetic material (DNA), interference in the cell cycle, affecting pH, disrupting the plasma membrane by increasing hydrophobic stress, phototoxicity, and formation of reactive oxygen species [74]. The effect of the pigment has been observed to vary according to if the bacteria is Gram-positive or negative. Prodigiosin is known to lyse Gram-positive bacterial cell walls leading to their death, while in the case of Gram-negative bacteria it affects gene expression and protein synthesis eventually altering the cellular life cycle and metabolism [24].

Prodigiosin has also been observed to have a toxic effect on certain fungal species, such as *Batrachochytrium dendrobatidis*, *B. salamandrivorans*, *Pythium myriotylum*, *Rhizoctonia solani*, *Sclerotium rolfsii*, *Phytophthora infestans*, *Fusarium oxysporum*, and *C. nymphaeae*. The pigment’s antimycotic activity could be of use to control or to eradicate parasitic fungal species. The most common parameter for comparing antifungal activity was percentage inhibition, which is equal to the percentage difference in fungal cells with and without the pigment. Table 3 contains different recent studies that report prodigiosin’s antimycotic effect.

### 4.2. Antioxidant

In the food industry, artificial additives are used to preserve food, which have shown deterioration in health due to their consumption, such as cell damage, inflammation, metabolic disorders, among others. Recently, an interest in natural preservative additives has been shown, given their benefit in human health. A balanced consumption of antioxidant compounds or foods rich in antioxidants allows for a reduction in oxidative stress and free radicals levels, generating protective effects in cells and balancing the immune system to produce defenses against various diseases, such as stress, cancer, hypertension, atherosclerosis, and gastrointestinal and hormonal disorders [80,81,82,83].

Prodigiosin has demonstrated high antioxidant potential with interest for many applications. The strong antioxidant activity may be attributed to conjugated double bond and ring pyrrole structures of prodigiosin [84]. A recent study demonstrated the high antioxidant activity of purified prodigiosin from *Serratia marcescens*, showing 92 and 99% of scavenging at 5 mg/mL of prodigiosin, for free radicals ABTS and DPPH, respectively. The antioxidant properties of prodigiosin also demonstrated benefits in the preservation of foods through the decrease of rancidity and microbiological contamination, increasing shelf life, and adding some additional properties, such as color for a pleasant appearance for the consumer [85]. Prodigiosin purified from radio-resistant *Streptomyces* sp. WMA-LM31 showed antioxidant activity for DPPH with a scavenging capacity of 62%, a protein oxidation inhibition of 54.8%, and a lipid peroxidation of 25.4% at 10 µg/mL, showing strong antioxidant activity at low concentrations [84]. Prodigiosin also showed strong activity against different free radicals, the ability to block the formation of superoxides, and the inhibition of Fenton reactions, reducing the negative effects of protein and lipid oxidation as well as the prevention of DNA damage [84]. Nguyen et al. produced prodigiosin from marine chitinous wastes by a bioprocess with *S. marcescens* strains, reporting a moderate antioxidant activity, with values of inhibition (IC_50_) of 115 and 235 µg/mL for ABTS and DPPH, respectively [83].

### 4.3. Antitumoral

Cancer is a global health problem, representing one of the main causes of mortality worldwide and the first or second of premature mortality in the main countries of the world. According to the World Health Organization, in 2020 an estimated 19.3 million new cases and 10 million deaths were estimated in which each of 5 people in the world developed cancer during their lifetime and in which one in 11 women and one in 8 men died from cancer [86,87]. For 2070, a strong increase in the incidence of cancer is estimated, twice the current level, due to population growth and aging, demographic changes and an increase in risk factors for cancer development associated with demographic increase [88]. The main types of cancer with the highest incidence in 2020 are breast, prostate, lung, and colorectal cancers, and the cancers with the highest mortality rates were lung, colorectal, and liver, in order of appearance [89]. The uncontrolled increase in the incidence of cancer, high costs of treatment, and resistance to current drugs have created a great need to design, research, and discover new compounds. The search for natural compounds produced by different organisms has been the most important strategy, highlighting that more than half of the approved treatments against cancer are of natural origin or derivatives [90].

Prodigiosin is the most important secondary metabolite from *Serratia marcescens*, with strong biomedical applications against therapeutic diseases at low concentrations, such as cancer, and showing a high apoptotic effect against cancer cells and low or no toxicity on normal cells, as shown in Table 4 [91]. Prodigiosin has shown a high potential as an antitumoral agent against colorectal cancer, inhibiting late-stage autophagy and increasing sensitivity to 5-fluorouracil of different colorectal cancer cells (HCT116 and SW480) through blocking autophagosome–lysosome fusion and maturation of lysosomal cathepsin [92]. Ji et al. demonstrated that prodigiosin markedly decreases the proliferation of K562 cells (chronic myelogenous leukemia) through increased activity of caspases-3, -8, -9 and increased reactive oxygen species, resulting in the inhibition of autophagy and the induction of apoptosis [93]. Nguyen et al. demonstrated the strong anticancer effect of prodigiosin in different cancer cell lines, MCF-7, A549, HepG2 and WiDr, with inhibition values of 92.1%, 93.1%, 94%, and 92%, and low values of IC_50_, 0.102 µg/mL, 0.182 µg/mL, 0.161 µg/mL, and 0.441 µg/mL, respectively [94]. The purified prodigiosin bioproduced from marine chitin showed high anticancer activity against MCF-7, A549, and HepG2, and an efficacy of 2.75, 1.67, and 3.25 times greater than Mytomycin C, a commercial anticancer compound, respectively, proposing some mechanisms that prodigiosin affects, such as mitogen-activated protein kinase regulators, pH modulators, DNA cleavage agents, and cell cycle inhibitors [95]

An anticancer study demonstrated the effect of prodigiosin through tests with prostate cancer (PC3) and human choriocarcinoma (JEG3) cell lines in vitro and PC3 and JEG3 tumor-bearing nude mice in vivo, showing an inhibition of the proliferation and a reduction in the size and weight of the tumors, depending on the prodigiosin concentration and the treatment time, respectively [96]. Berning et al. demonstrated that prodigiosin increases the sensitivity of cisplatin-resistant and sensitive urothelial carcinoma cell lines (RT-112) to cisplatin mediated by dysregulation of lysosomal function and reduction of cathepsin B and L activity [23]. Prodigiosin has great cytotoxic activity against many melanoma cancers cells lines, such as NGM, 501-Mel, WM293A, HT-144, SK-Mel-19, SK-Mel-28, and SK-Mel-147, showing mean IC_50_ values of 0.2 µM through kinases modulation, intracellular acidification, DNA damage, and apoptosis induction [97]. Breast cancer is the type of cancer with the highest incidence worldwide and the second with the highest mortality rate. Prodigiosin has recently shown very relevant effects in the fight against this disease. In MDA-MB-231 (hormone-independent breast cancer cell line) and MDA-MB-468 cells, the prodigiosin at nanomolar concentrations, blocking the Wnt/β-catenin pathway, decreased phosphorylation of GSK3β, DVL2, and LRP6 and suppressed β-catenin–stimulated Wnt target gene expression [98].

A novel study of antitumoral activity of prodigiosin combined with PU-H71 against MDA-MB-231 showed high levels of caspases 3, 8, and 9 and decreased the levels of mTOR expression and HSP90α expression and transcription levels, which resulted in a breakthrough for a new therapy against triple negative breast cancer [99]. The high antitumor activity of prodigiosin at low concentrations and the low secondary effects against healthy cells has strengthened and intensified research into it as a new anticancer therapy. Obatoclax, a synthetic analog of prodigiosin, is being studied in phase I and II clinical trials for the treatment of lymphoma, myelodysplastic syndrome, and lung cancer [97].

**Table 4:** Antitumoral activity of prodigiosin against different cancer cell lines.

Cancer Type	Cell Line	Mechanism	IC_50_	Units	Reference
Blood/Leukemia	K562	Increased activity of caspases -3, -8, -9, reactive oxygen species, inhibition of autophagy and apoptosis induction.	>500	µM	[93]
	HL-60	Apoptosis induction	1.7	µg/mL	[100]
	Wt-p53Molt-4	Caspase-3-dependent apoptosis	1.3	µM	[101]
Brain	GBM8401	ER stress/autophagy	7.36	µM	[102]
	U87MG	ER stress/autophagy	12.29	µM	
Breast	MDA-MB-231	ER stress; Wnt/β-catenin; JNK/MAPK/RAD51	62.52	nM	[98]
	MDA-MB-468	Inhibit Wnt/β-catenin	261.2	nM	
	MCF-7	Apoptosis induction	5.1	µg/mL	[100]
	MCF-7	Mitogen-activated protein kinase regulators, pH modulators, DNA cleavage agents and cell cycle inhibitors	0.04	µg/mL	[94]
	MDA-MB-231	Decreased the levels of mTOR and HSP90α expression and transcription	2.1	nM	[99]
Urothelial	RT-112	Dysregulation of lysosomal function and reduction of cathepsin B and L activity	675	nM	[23]
Colorectal	DLD1	c-Jun/ΔNp73/p73/apoptosis; Lysosomal acidification	>1.6	µM	[103,104]
	HCT116	c-Jun/ΔNp73 p73 activation	4	µM	
	SW480	c-Jun/ΔNp73 p73 activation		µM	
	SW-620	Apoptosis	0.273	µM	
	HCT116; SW480	Blocking autophagosome–lysosome fusion and maturation of lysosomal cathepsin	>0.1	µM	[92]
	WiDr	Mitogen-activated protein kinase regulators, pH modulators, DNA cleavage agents and cell cycle inhibitors	0.05	µg/mL	[97]
Liver	HepG2	Antiproliferative effects	12.64	µg/mL	[84]
	HepG2	Mitogen-activated protein kinase regulators, pH modulators, DNA cleavage agents and cell cycle inhibitors	0.04	µg/mL	[95]
Lung	A549	PI3K-p85/Akt/mTOR; PKB/SKP2/p27	10	µM	[105]
	CNE2, NP69	PKB/SKP2/p27	4 and 0.35	mg/L	[106]
	NCHI-292	Apoptosis induction	3.6	µg/mL	[100]
	A549	Mitogen-activated protein kinase regulators, pH modulators, DNA cleavage agents and cell cycle inhibitors	0.06	µg/mL	[95]
Prostate	PC3	Intrinsic apoptosis	>10	µg/mL	[96]
Trophoblast	JEG3	Intrinsic apoptosis	>10	µg/mL	
Uterus	Hela	Antiproliferative effects	12.75	µg/mL	[84]
Gingival squamous carcinoma	OECM1	Cyclin D1 inhibition, arresting cell cycle in G0/G1 phase	1.59 ± 0.77	μM	[107]
Tongue	SAS	cyclin D1 inhibition, arresting cell cycle in G0/G1 phase	3.25 ± 0.49	μM	
Uterus	Hela	Intrinsic apoptosis	0.5–2.1	μg/mL	[108]

### 4.4. Antiprotozoal

Natural compounds and their semisynthetic derivatives are the primary strategy for obtaining and developing new drugs for the treatment of parasitic diseases [109]. Malaria is a disease that threatens global health, as it has developed resistance to current drugs and is causing more than a million deaths annually. Prodigiosin and derivatives, such as undecylprodigiosin and metacycloprodigiosin in nanomolar concentrations and IC_50_ between 5–12 nM, were shown to exhibit potent in vitro antimalarial activity against Plasmodium falciparum, the parasite that causes malaria disease in humans [110,111]. Mosquitoes are the vectors for the transmission of deadly diseases, such as malaria and dengue. The control of the reproduction and spread of mosquitoes is an alternative for the control and reduction of new infections [112]. The larvicidal activity of prodigiosin has been reported, and it is known. Prodigiosin at a concentration of 100 ppm demonstrated a larvicidal activity against *Aedes aegypti* of 32% and 76% mortality with 24 and 48 h of incubation, respectively [113]. Purified prodigiosin has shown high larvicidal activity against larval and pupal stages of *Aedes aegypti* and *Anopheles stephensi* mosquitoes, obtaining IC_50_ values between 14 to 21 µg/mL and 19 to 32 µg/mL, respectively, in the various growth stages. The larvicidal concentration to both mosquitoes was found at 62.5 µg/mL for the three firsts growth stages [112].

### 4.5. Antiviral

Herpes Simplex Virus (HSV) is one of the most contagious infections worldwide. Both are lifelong, and sometimes it can cause painful blisters or ulcers at the site of the infection. Approximately 491.5 million people had HSV type 2 infection in 2016, and approximately 3752 million people were living with HSV type 1, this is equivalent to 79.4% of the world’s population [113,114]. This virus can also affect eye tissue and cause keratitis, eye drops are commonly used for medical treatment of this infection. In some cases, surgical treatment may be necessary to treat complications [115]. A recent study demonstrated the antiviral effect of prodigiosin on HSV type 1 and HSV type 2 infections through in vitro and ex vivo cultured mice corneas. Prodigiosin treatment significantly protects the eyes of mice, reducing the disease’s development; it also protects against the loss of corneal sensitivity and excessive inflammation, showing nontoxic effects [115]. At non-toxic concentrations, prodigiosin exhibited significant in vitro antiviral activity against cells infected with *Bombyx mori* nucleopolyhedrovirus (BmNPV). The result of prodigiosin action was selective death of infected cells inhibiting viral gene transcription (*ie-1*) and preventing virus-mediated membrane fusion, resulting in inhibition of virus production and replication [116].

## 5. Recent Advances and Applications of Prodigiosin Bionanocomposites

Prodigiosin is a microbial metabolite with numerous bioactivities and many possible applications in biomedicine for the treatment of many diseases. However, the effectiveness of prodigiosin in clinical treatment is limited by its poor absorption, hydrophobicity, and low bioavailability [117]. Nanoscale drug carriers are an attractive biotechnology to overcome the limitations of the use of prodigiosin, which allows for obtaining formulations with high functional yields for target delivery systems in the treatment of cancer [118]. A study on the binding of prodigiosin to organic and inorganic matrices represents significant importance to improving the use of prodigiosin as a bioactive compound in applied pharmacology [119]. Natural biomolecules, such as starch, proteins, biopolymers, and chitosan, are an attractive group of scaffold compounds compared to the synthetic, due to their low toxicity and biodegradability [120,121]. Nanoparticle technology utilized several benefits of biopolymer scaffolds carrying hydrophobic ligands to target sites. An essential step to design biopolymer-based nanoparticle systems is to understand the binding sites and binding affinity of the protein-drug complex [122]. Although previous studies have shown the interaction potential of prodigiosin (or pirrolyc) with some biopolymers or proteins, such as bovine hemoglobin and bovine serum albumin, to the best of our knowledge, there is no report detailing the interactions of chitosan, PLA, and other polyacids. A biodegradable formulation of PLGA-based microparticles loaded with a bacterial prodigiosin showed an antitumor activity against a triple negative breast tumor, reducing the cell viability of the MDA-MB-231 cell line, being a new proposal for the controlled application of the prodigiosin as a drug for cancer treatment [123]. A promising prodigiosin nanocarrier as a hybrid system of nanocomposite of β-cyclodextrin and chitosan with nanoparticles improve the activity and selectivity against cancer cells (MCF-7 and HepG2 cell lines), compared with free prodigiosin, and showed no toxicity against healthy cells (NIH/3T3 cells) [124]. The localized and controlled administration of chemotherapeutic drugs was demonstrated by hybrid nanofiber systems composed of PLGA, Pluronic F127, gelatin, and prodigiosin as active compounds for the treatment of triple negative breast cancer, decreasing cell viability and inducing apoptosis cells in MCF-7 and MDA-MB-231 cell lines [125]. Another recent study showed nanocomposites that increase the bioavailability of prodigiosin for use as a cytotoxic drug against *Caco-2* and HCT116 carcinoma cell lines, without negative effects in healthy cells [118].

## 6. Concluding Remarks and Futures Perspectives

Prodigiosin is a bioactive compound that is obtained from the secondary metabolism of bacteria and can easily be produced at the laboratory level and scaled up to bioreactors, as well as generating strategic genetic modifications to increase its production to use prodigiosin in different biotechnological applications. The bioactivity of prodigiosin is known and its positive effects have been described and accumulated over time in different in vitro models for antioxidant, antimicrobial, anticancer, antiviral, and antiprotozoal activity, highlighting its antimicrobial and anticancer activity due to its high efficiency at low concentrations against different cancer cell lines and not presenting any negative effect against healthy cells. However, the low bioavailability and low absorption of prodigiosin are some problems that can be overcome with the prodigiosin nanocarriers strategy.

The development of prototypes of prodigiosin functionalized bionanocomposites is the most recent strategy to potentiate and to obtain new carriers for the transport and controlled release of prodigiosin—which are not normally achieved with free prodigiosin—and thus achieve the desired bioactivity, however, new studies in vivo are required to obtain new information regarding its safety and effectiveness.

## Figures and Tables

**Table 1:** Prodigiosin producing bacteria reported in literature.

Microorganism (Reference)	Origin	Prodiginine Type	Microorganism (Reference)	Origin	Prodiginine Type
*Serratia marcescens UCP 1549* [20]	Semi-arid soil around banana trees	Prodigiosin	*S. coelicolor M1110* [48]	N/A	Undecylprodigiosin
*Streptomyces sp. JS520* [26]	Pristine soil sample collected from the cavy area of Miroc mountain in eastern Serbia	Undecylprodigiosin	*S. marcescens SMAR* [49]	N/A	Undecylprodigiosin
*S. marcescens Xd-1* [45]	From a mold and tofu sample	Prodigiosin	*Actinomadura sp* [50]	Sediment samples in Brazil	Nonylprodigiosin Cyclononylprodigiosin Methylcycloctilprodigiosin
*S. coelicolor M145* [46]	N/A	Undecylprodigiosin	*S. marcescens* [51]	From rhizospheric soil in different sites of Salem and Namakkal, India	Prodigiosin (possible)
*S. parvulus* [52]	N/A	Butylcycloheptylprodigiosin and Undecylprodigiosin	*S. marcescens NCIM 5061* [53]	N/A	Prodigiosin
*S. nematodiphila RL2* [54]	From Lahul and Spiti region in Himachal Padresh, India	Prodigiosin	*S. marcescens ATCC 13880* [55]	N/A	Prodigiosin
*S. rubiaea RAM Alex* [56]	From clam samples collected form Temsah Lake, Egypt	Prodigiosin	*S. marcescens* [57]	From kitchen waste	Prodigiosin (Possible)
*S. marcescens* [58]	Soil sample from a tannery in Chennai, Tamilnadu, India.	Prodigiosin	*S. spectabilis BCC 4785* [59]	From soil sample collected in Thailand	Metacycloprodigiosin

N/A: not applicable.

**Table 2:** Recent antibacterial activity of prodigiosin reported in literature.

Prodigiosin Source	Concentration	Bacteria	Parameter of Bactericide Action	Value	Reference
*Serratia rubidaea* RAM_Alex	Chiffon stained by prodigiosin	*Escherichia coli*	AATCC 100 Bacteria reduction (%)	95	[24]
		*Staphylococcus aureus*		97	
	Satin stained by prodigiosin	*Escherichia coli*		91	
		*Staphylococcus aureus*		84	
	Linen stained by prodigiosin	*Escherichia coli*		97	
		*Staphylococcus aureus*		70	
	Dacron stained by prodigiosin	*Escherichia coli*		90	
		*Staphylococcus aureus*		84	
	Gabardine stained by prodigiosin	*Escherichia coli*		19	
		*Staphylococcus aureus*		15	
*Serratia marcescens*	Silk pH 2.1	*Staphylococcus aureus*	Antibacterial rate	93.17%	[75]
		*Escherichia coli*		25.12%	
	Silk pH 8.1	*Staphylococcus aureus*		87.80%	
		*Escherichia coli*		14.70%	
*Serratia marcescens*	25–400 µg/mL	*Staphylococcus aureus*	IC_50_	51.17 µg/mL	[71]
		*Listeria monocytogenes*		51.54 µg/mL	
		*Enterococcus faecium*		26.18 µg/mL	
		*Bacillus cereus*		33.61 µg/mL	
		*Salmonella enteritidis*		56.56 µg/mL	
		*Proteus vulgaris*		50.81 µg/mL	
		*Pseudomonas aeruginosa*		69.71 µg/mL	
		*klebsiella pneumoniae*		48.63 µg/mL	
		*Aeromonas hydrophila*		66.98 µg/mL	
		*Escherichia coli*		44.20 µg/mL	
		*E. coli O157:H7*		20.31 µg/mL	
*Achromobacter denitrificans SP1*	25 µg/mL	*Proteus mirabilis*	Maximum zone of inhibition (mm)	17	[72]
		*Staphylococcus aureus*		15	
*Serratia marcescens*	250 μg/mL	*MRSA*	Maximum zone of inhibition (mm)	20 ± 0.33	[74]
		*Staphylococcus aureus*		20 ± 0.0	
		*Enterococcus faecalis*		20 ± 0.88	
		*Escherichia coli*		22 ± 0.41	
	500 μg/mL	*MRSA*		21 ± 0.00	
		*Staphylococcus aureus*		22 ± 0.33	
		*Enterococcus faecalis*		20 ± 0.33	
		*Escherichia coli*		27 ± 0.82	
*P. putida strain pig-r2*	24.48 μg/mL	*Corynebacterium glutamicum*	Minimal inhibitory concentration (MIC)	2.56 μg/mL	[73]

**Table 3:** Antifungal activity of prodigiosin.

Prodigiosin Source	Concentration	Fungi	Parameter of Antifungal Action	Value	Reference
*Serratia plymuthica and S. marcescens*	3.8 µM	*Batrachochytrium dendrobatidis (Bd)*	IC_50_	3.8 µM	[76]
	27.3 μM	*B. salamandrivorans (Bsal)*	IC_50_	27.3 μM	
	10 μM	*Batrachochytrium dendrobatidis (Bd)*	Minimum inhibitory concentration (MIC)	10 μM	
	50 μM	*B. salamandrivorans (Bsal)*	Minimum inhibitory concentration (MIC)	50 μM	
*Serratia sp.*	Crude extract from rhizosphere of *Bacopa monnieri* (L.)	*Pythium myriotylum*	Percent inhibition	71.33	[77]
		*Rhizoctonia solani*	Percent inhibition	61.33	
		*Sclerotium rolfsii*	Percent inhibition	49.33	
		*Phytophthora infestans*	Percent inhibition	48.66	
		*Fusarium oxysporum*	Percent inhibition	31	
*Serratia rubidaea Mar61-01*	450 µg/mL	*C. nymphaeae*	Percent inhibition	29.27	[78]
	1000 µg/mL	*C. nymphaeae*	Percent inhibition	100	
*Serratia spp. isolated from medicinal plant Plumbago indica*	-	*Pythium myriotylum*	Percent inhibition	40	[79]

## Data Availability

Not applicable.
